# Targeting the Synapse in Alzheimer’s Disease

**DOI:** 10.3389/fnins.2019.00735

**Published:** 2019-07-23

**Authors:** Johanna Jackson, Enrique Jambrina, Jennifer Li, Hugh Marston, Fiona Menzies, Keith Phillips, Gary Gilmour

**Affiliations:** ^1^Lilly Research Centre, Eli Lilly and Company, Windlesham, United Kingdom; ^2^Lilly Research Laboratories, Eli Lilly and Company, Alcobendas, Spain

**Keywords:** synapse, plasticity, drug discovery, translational research, biomarker

## Abstract

Dynamic gain and loss of synapses is fundamental to healthy brain function. While Alzheimer’s Disease (AD) treatment strategies have largely focussed on beta-amyloid and tau protein pathologies, the synapse itself may also be a critical endpoint to consider regarding disease modification. Disruption of mechanisms of neuronal plasticity, eventually resulting in a net loss of synapses, is implicated as an early pathological event in AD. Synaptic dysfunction therefore may be a final common biological mechanism linking protein pathologies to disease symptoms. This review summarizes evidence supporting the idea of early neuroplastic deficits being prevalent in AD. Changes in synaptic density can occur before overt neurodegeneration and should not be considered to uniformly decrease over the course of the disease. Instead, synaptic levels are influenced by an interplay between processes of degeneration and atrophy, and those of maintenance and compensation at regional and network levels. How these neuroplastic changes are driven by amyloid and tau pathology are varied. A mixture of direct effects of amyloid and tau on synaptic integrity, as well as indirect effects on processes such as inflammation and neuronal energetics are likely to be at play here. Focussing on the synapse and mechanisms of neuroplasticity as therapeutic opportunities in AD raises some important conceptual and strategic issues regarding translational research, and how preclinical research can inform clinical studies. Nevertheless, substrates of neuroplasticity represent an emerging complementary class of drug target that would aim to normalize synapse dynamics and restore cognitive function in the AD brain and in other neurodegenerative diseases.

**GRAPHICAL ABSTRACT:**
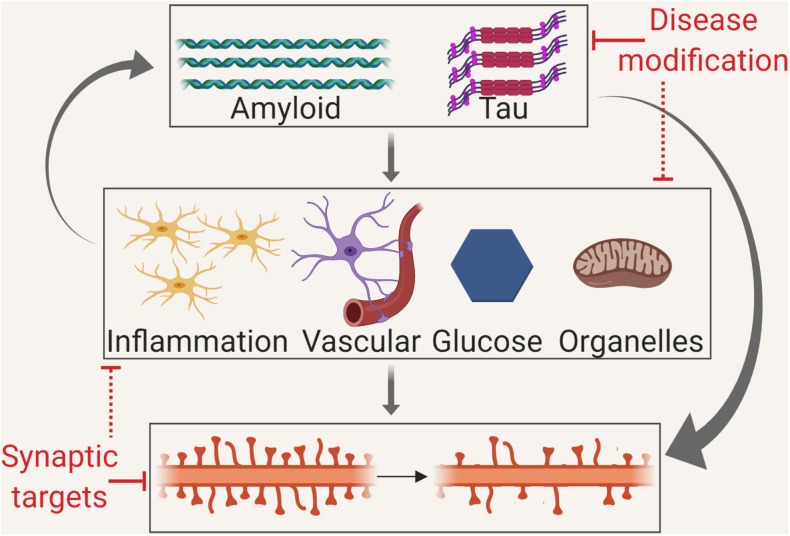
Disruption of neuronal plasticity may occur via several mechanisms. Therefore, the modulation of plasticity may be by disease modification targeting misfolded proteins, by targeting the intermediate mechanisms or by direct synaptic therapies.

## Concepts of Plasticity

**FIGURE 1:**
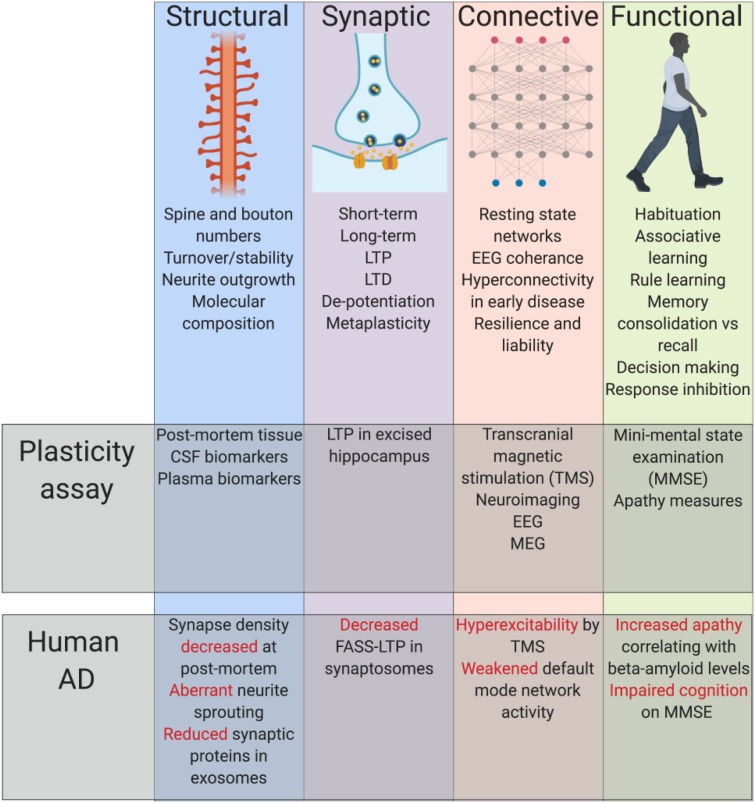
Summary of established principles. Plasticity can be conceptualized and defined at several different levels of function, from basic biochemical events to integrated behavioral responses, each of which have shown impairment alongside classical AD pathology. Cerebral spinal fluid (CSF), long-term potentiation (LTP), long-term depression (LTD), electroencephalogram (EEG), magnetoencephalogram (MEG), fluorescence analysis of single-synapse LTP (FASS-LTP).

At the most general level, plasticity refers to the ability of the nervous system to dynamically modulate its function in response to ongoing internal activities or external experiences. Plasticity is a normal and essential part of cognition, also an important means by which the brain can respond to damage. Plasticity can be conceptualized and defined at several different levels of function, from basic biochemical events to integrated behavioral responses (Figure 1). Structural plasticity relates to the physical morphology of synapses. The term synaptic plasticity *per se* is most typically used to describe processes related to the efficiency of transmission across new or existing synapses. A fundamental concept linking structural and synaptic plasticity is Hebbian learning (Hebb, 1949), where a threshold of coincident activity at synapses between cells will result in relative strengthening of these connections, potentially balanced by the relative weakening of other connections. Hebbian plasticity is a key substrate of a variety of different learning events. It is complemented by processes of homeostatic plasticity, mechanisms that rescale synaptic numbers and efficiencies to maintain an overall stable synapse density. At a more macroscopic level, connective plasticity can be described where groups of neurons adjust their means and patterns of communication between each other. All these processes contribute toward the expression of functional plasticity, an observable and flexible change in the behavioral repertoire and/or cognitive capacity of an animal.

## Measures of Neuroplastic Deficiency in AD

Synapse loss and dysfunction is a key feature in many neurodegenerative diseases including dementia and, in particular, AD. The idea that AD is principally a disorder of synaptic function, i.e., a “synaptopathy,” is by no means new (Selkoe, 2002). Late stage AD involves an incontrovertible and substantial loss of neurons and synapses (Jack et al., 2010). Of more interest is the possibility that synaptic dysfunction can occur early in prodromal or MCI stages of the disease and may robustly present before frank atrophy (Scheff et al., 2006). Currently, there is no validated *in vivo* biomarker that can be used to directly measure synaptic integrity over time in patients. Instead, synaptic dysfunction is inferred from the measurement of several different parameters in AD patients. The most direct method is neuroanatomical, to directly count the number of synapses present in defined brain regions and such studies have produced interesting findings (Scheff et al., 2006). However, neuroanatomy studies come fraught with confounds and artefacts related to post-mortem tissue sample work, can be prone to selection bias, and offer only a snapshot of pathology often at the end stages of disease. Along similar lines, there is a large and growing literature assessing indirect proxies of synaptic integrity, most typically by measuring abundance of mRNA or synaptic protein (Masliah et al., 2001). Care must be taken not to conflate loss of synaptic protein or message markers with actual loss of functional synapses themselves unless this has been directly validated. Other evidence of synaptic dysfunction can be inferred from electrophysiological and neuroimaging studies. For instance, *ex vivo* measurement of synaptic transmission parameters from brain sections from patients who have undergone surgical resection is possible, and toxic effects of beta-amyloid oligomers are reported in this context (Mendes et al., 2018). Quantitative EEG and MEG studies suggest alterations to synaptic function at various stages of the disease (Hulbert and Adeli, 2013). Neuroimaging studies suggest important effects of disease pathology on measures of functional connectivity. In this regard, default mode network activity seems to be disproportionately weakened in the early stages of AD possibly due to the regionally specific effects of amyloid pathology on synapses in key nodes of this network (Sperling et al., 2009).

## Evidence for Amyloid and Tau Driven Changes in Synaptic Plasticity

The two major histopathological hallmarks of AD are β-amyloid and hyperphosphorylated tau protein species, which can accumulate to form extracellular amyloid plaques and intracellular neurofibrillary tangles, respectively. β-amyloid plaque deposition *per se* does not seem to bear a strong relationship to synaptic dysfunction and loss in AD although soluble oligomeric species may play a more significant role. Evidence from J20 amyloid mice suggests that levels of synaptophysin puncta are decreased and negatively correlate with soluble β-amyloid protein, but not plaque load (Mucke et al., 2000). Synapse loss close to plaques may therefore be caused by increased levels of soluble oligomeric species rather than the plaques themselves (Koffie et al., 2009). Mechanistically, β-amyloid has been proposed to be a potential binding partner of a range of synaptic proteins. Arguably, the most important genetic risk factor described for AD is the apolipoprotein E4 gene, which has been implicated in facilitating the transport of oligomeric β-amyloid species to synapses where they can have toxic effects (Koffie et al., 2012). Soluble β-amyloid can also increase levels of DKK1, an endogenous wnt pathway antagonist that can activate non-canonical signaling to cause localized cell loss and synapse disassembly (Purro et al., 2012). In contrast to β-amyloid, patterns of tau hyperphosphorylation much more closely track cognitive decline in AD patients (Jack et al., 2010), suggesting a more critical role in synapse dysfunction. While neurofibrillary tangles may be an ultimate hallmark associated with neurodegeneration in AD, no evidence exists to suggest that tangles directly affect synaptic density in dysfunctional neurons (Rocher et al., 2010). Soluble, non-aggregated forms of tau may be more causally related to synaptic dysfunction than tangles are. Tau is normally present in synapses of both healthy and AD brains, with there being a greater relative level of hyperphosphorylated species present in the AD synapse (Fein et al., 2008). Tau species are also well described to be able to spread trans-synaptically between neurons in the AD brain (Ahmed et al., 2014), suggesting an intimate link between tau propagation, disease progression, and synaptic function. Normal tau also plays an important role in cellular transport as a microtubule stabilizing protein, and pathological tau species have been associated with impaired axonal transport in post-mortem AD brains (Dai et al., 2002). Tau-induced impairments in axonal transport are hypothesized to disrupt the normal transport of organelles such as mitochondria, which are essential for healthy synapse function (Mandelkow et al., 2003). This idea is supported from findings of ultrastructual post-mortem analyses, where more abnormal mitochondria are found in synapses of human AD brains compared to healthy controls (Pickett et al., 2018). As well as compromising mitochondrial transport and dynamics, pathological tau species can often be mislocalized to the somatodendritic compartment of cells (Hoover et al., 2010), and other post-synaptic sites. Such mislocalization can potentially result in further molecular pathologies, such as increases in fyn-tau interactions (Bhaskar et al., 2005) and AMPA receptor clustering deficits (Miller et al., 2014), both of which may further compromise synapse integrity. One other potentially important interaction in this context is that between β-amyloid and tau itself. Approximately one third of synapses in AD patients demonstrate colocalization of β-amyloid and tau (Fein et al., 2008). A recent study has shown that a large number of synaptic proteins, including tau, are phosphorylated in the presence of β-amyloid (Wu et al., 2018). Such work might suggest a potential pathological sequence whereby tau is more likely to become hyperphosphorylated in the presence of β-amyloid, and the greater the level of hyperphosphorylated tau in the synapse, the greater the probability of synaptic loss and thereby symptoms occurring.

β-amyloid and tau may also have other more indirect effects on synaptic function via for instance inflammatory processes or disturbances to glucose homeostasis. Considering inflammation, the presence of misfolded proteins in the AD brain can trigger an innate immune response, release inflammatory mediators, and increase levels of activated microglia, all of which can contribute to disease progression and severity (Heneka et al., 2015). Genome wide association studies have shown that genes with roles in innate immunity are risk factors for the development of AD (Lambert et al., 2009). The importance of local neuroinflammatory processes in AD was recently highlighted by discovery of a genetic mutation in TREM2 that significantly increases the risk of AD (Guerreiro et al., 2013). TREM2 is involved in phagocytic clearance of neuronal debris, and such clearance mechanisms may provide a link between inflammation, microglial activity and synaptic integrity. In AD mouse models, activated microglia have been shown to engulf and excessively prune synapses via a complement-dependent mechanism, resulting in synapse loss (Hong et al., 2016). Furthermore, activated microglia release pro-inflammatory cytokines such as TNFα and IL-1β which can have direct excitotoxic effects on synapses (Wang et al., 2015). Systemic inflammation, as caused by infection or injury, may exacerbate local neuroinflammatory processes. It is well known that systemic inflammation is associated with the development of cognitive deficits in the elderly, which may possibly be a consequence of “primed” microglia producing pro-inflammatory cytokines after receiving a trigger from the periphery (Dilger and Johnson, 2008). Systemic inflammation is also thought to lead to the production of β-amyloid, which is suggested to have antimicrobial properties (Soscia et al., 2010). In an AD patient where the blood brain barrier is already more permeable than normal (Montagne et al., 2015), it is quite possible that exposure to systemic inflammation could result in a heightened inflammatory response which would further expedite synapse loss. A second important mechanism by which synaptic integrity may be compromised in AD relates to insulin signaling. Not only does insulin signaling play a critical role in synaptogenesis during development (O’Kusky et al., 2000), but insulin plays a role in the maintenance of learning and memory processes in the adult brain (Moosavi et al., 2006). Regarding pathological states, it has been hypothesized that obesity can cause a central insulin resistance that contributes to synaptic plasticity deficits. This is evidenced by decreases in PSD-95 protein in dendritic spines and ultimately a greater presentation of cognitive impairments in obese subjects (Arnold et al., 2014). In mouse models, intrahippocampal administration of β-amyloid oligomers impairs insulin signaling and hippocampal metabolism, which was associated with an impairment of spatial working memory (Pearson-Leary and McNay, 2012). In human post-mortem tissue, cells with hyperphosphorylated tau inclusions have been found to also contain oligomeric insulin accumulations. Insulin accumulations track the progression of tau pathology in these tissues, suggesting potential for a mechanistic relationship between these species (Rodriguez-Rodriguez et al., 2017).

Whilst the exact mechanisms of amyloid and tau-induced synapse loss are still not fully understood, there are many possible direct mechanisms and also several indirect mechanisms that are likely to play a significant role, such as inflammation and insulin resistance. All these processes will interact to produce pathological synaptic dysfunction and define the symptomatic trajectory for individual patients.

## Approaches Toward Therapeutic Intervention

Therapeutic interventions focussed on the treatment of synaptopathy in AD could be targeted at the theoretically distinct processes of maintenance, compensation and recovery of synaptic function (Sheng et al., 2012). Given that AD is a disease predominantly described by its impact on cognitive symptoms, a topic of significant interest has been the potential for cognitive training to maintain or support performance capacity. Recent meta-analyses (Hill et al., 2017; Carrion et al., 2018) suggest that there may well be important positive findings here, although work related to AD could be improved with the use of more randomized controlled trials with better specification of the cognitive domains under assessment. It may be that the most impactful synaptic efficacy could be offered by the combination of cognitive training and pharmacotherapy.

**FIGURE 2:**
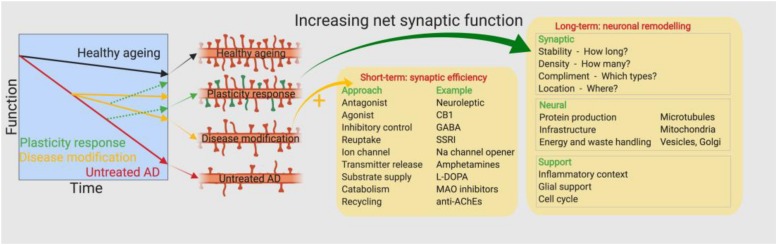
Approaches toward therapeutic intervention. Earlier drug discovery efforts pursued a maintenance approach by attempting to improve the efficiency of remaining synapses in the AD brain, however, the approved drugs have limited efficacy depending on the magnitude and stage of the disease. A new approach is required to target the ongoing synapse loss and restore the numbers of functional synapses.

The earliest drug discovery efforts pursued a maintenance approach by attempting to improve the efficiency of remaining synapses in AD brains. Pharmacological classes here include the anti-cholinesterases (Anand and Singh, 2013), selective serotonin reuptake inhibitors (Chow et al., 2007) and NMDA antagonists (Prentice et al., 2015; Figure 2). While cholinesterase inhibitors and the NMDAR antagonist memantine are approved for treatment of AD, their efficacies are somewhat limited in magnitude and by disease stage (Tampi and van Dyck, 2007). This limited efficacy may be the result of the inability of such agents to therapeutically address ongoing synaptic loss. Another strategic approach might be to attempt to further promote endogenous mechanisms of compensation in the AD brain. The healthy brain has a great ability to compensate for synapse loss caused by different neurological diseases or insults. Overt symptoms are not always observed following significant brain damage, such as tumours or strokes. In AD, processes of compensation may explain the delay between initial signs of synaptic loss and the clinical presentation of memory deficits (Katzman, 2004). The period for which the brain can engage compensatory mechanisms and remain resilient to injury may depend to a significant degree on its pre-injury state; the so-called theory of “cognitive reserve” (Stern, 2012). Computational studies modeling power spectra and synapse loss demonstrate that processes of local and global compensation are able to maintain power spectrum dynamics despite frank synapse loss (Abuhassan et al., 2014). Compensatory mechanisms could include increases in the size of remaining synapses (Scheff, 2003), changes in synapse dynamics (Jackson et al., 2017) or changes in synaptic connectivity parameters within distributed neuronal networks (Abuhassan et al., 2014). Boosting the brain’s endogenous compensatory capacity might be a relevant therapeutic strategy (Baazaoui and Iqbal, 2018) to mitigate synapse loss, at least in the early stages of AD. Changes in several neurotrophic factors, such as NGF, BDNF, and VGF, have been reported in AD patients. BDNF and VGF levels have been shown to be consistently decreased in several brain regions (Ferrer et al., 1999; Cocco et al., 2010), with less robust and more regionally dependent changes being found for NGF (Mufson et al., 1995). Administration of neurotrophic factors or analogs thereof presents considerable challenges from a drug development perspective, although some clinical and preclinical studies have been reported but with limited success thus far. A Phase 1 trial of three AD patients showed that direct intracerebroventricular infusion of NGF offered slight neuropsychological improvements, although side effects related to route of administration were observed (Mandel, 2010). In an amyloid precursor protein mouse mutant model, viral vector administration of BDNF resulted in a significant recovery of synapses, without altering neuronal cell numbers (Nagahara et al., 2009). Finally, administration of truncated ciliary neurotrophic factor in amyloid and tau mouse models restored synaptic loss and concomitant behavioral deficits (Kazim et al., 2014), however, treating outside the hypothetical compensation window may also yield similar effects (Bolognin et al., 2014). Novel delivery methods, such as encapsulated cell biodelivery, are emerging which may overcome some of the challenges associated with administering neurotrophic support in humans (Eyjolfsdottir et al., 2016). Such findings may suggest that distinctions between processes of compensation versus those of recovery may be less in practice than theory might suggest. In addition to neurotrophic mechanisms, the wnt pathway is a critical effector of synaptic integrity. Wnt pathway signaling is undoubtedly complex, although several studies have shown that targeting this pathway at a number of different places, such as blocking effects of ROCK (Sellers et al., 2018) or DKK-1 (Marzo et al., 2016), can have positive effects in restoring lost synapses. Identifying the most relevant biochemical networks supporting processes of synaptic plasticity, and then determining what the most druggable aspects of these networks are will be key to success here.

## Gaps and Issues in Translational Research

Current knowledge of synaptic changes in AD is derived predominantly from preclinical work, which may be problematic as no animal model can completely recapitulate the human disease state. Wherever possible, studies concerned with the validation of novel targets related to synaptic plasticity should prioritize study of human-derived tissue (i.e., biofluids, excised tissue, or iPSCs), or consider what possibilities there are to study synaptic plasticity non-invasively in living humans. Preclinical studies are still valuable – but should be utilized more in the context of understanding pharmacokinetic-pharmacodynamic relationships of drug effects rather than being expected to predict drug efficacy.

From an imaging perspective, volumetric MRI offers a very robust method to longitudinally measure the progression of atrophy in an AD brain, however, the technique is not likely sensitive enough at present to inform on subtle changes in synaptic dynamics that may be happening early in the disease. FDG PET imaging offers an alternative approach that might provide more information about synaptic activity (Segobin et al., 2015). Imaging studies have suggested that synapse dysfunction, as measured by FDG PET, occurs prior to gross atrophy, as measured by volumetric MRI (Jack et al., 2010). The resolution of FDG PET is still likely to be lacking, although such work may be useful to determine which early stage patients might benefit most from a synaptic therapy. To increase the spatiotemporal resolution of synaptic imaging methods, trials are underway to develop PET tracers that can reliably measure synapse density. The most advanced work here focuses on the presynaptic vesicle protein SV2a, and initial work suggests ligands like UCB-J show promise for the ability to measure synapse density over time (Finnema et al., 2016). Other electrophysiological and fluid biomarkers of synaptic function are also being considered. Electrophysiological biomarkers in development include quantitative EEG measurements, which may inform on synaptic activity and correlate with AD markers (Smailovic et al., 2018); rapid eye movement sleep patterns, which may be affected in AD (Reynolds et al., 1990); and event related potentials, which may index synaptic compensation processes in early AD (Chapman et al., 2013), although these are yet to be used routinely in the clinic. Likewise, CSF biomarkers such as neurogranin (Kester et al., 2015) and SNAP-25 (Brinkmalm et al., 2014) have shown promising relationships with synapse loss *in vivo* but have yet to be used routinely. Excitingly, recent work has shown that synaptic protein markers are secreted in exosomes and their levels are correlated to cognitive decline in FTD and AD (Goetzl et al., 2016).

Currently there are only three publicly disclosed drug trials with endpoints that specifically inform on synapse density and/or function. The LUCIDITY trial (NCT03446001) employs FDG-PET and cognitive endpoints to assess the effects of a methylene blue derivative on synaptic function. A second trial (NCT02167256) is also using similar FDG-PET and cognitive endpoints to assess the effects of the Src/Abl tyrosine kinase inhibitor saracatinib on synaptic function. Lastly, the SV2a PET ligand UCB-J is being used to measure the effects of the sigma2 receptor antagonist CT1812 on synaptic density in mild to moderate AD patients (NCT03493282). Hopefully positive findings in one or more of these studies will catalyze further research here.

## Outlook

Recent work has greatly advanced our understanding of the direct and indirect means by which β-amyloid and tau pathologies may negatively impact synaptic function in AD. Considering AD as a disease of synaptic function and finding novel therapies that specifically target the synapse is an exciting avenue of research that complements traditional approaches in this field. Discovery and validation of synaptic therapies should focus on human disease rather than animal models wherever possible. An initiative taking such an approach is the Accelerating Medicines Partnership – Alzheimer’s Disease (AMP-AD) network, which via post-mortem gene expression and proteomic studies in AD patients, has flagged several synapse-related gene networks. These include a VGF-centered Alzheimer’s disease network, where the majority of genes regulate neuronal activity, synaptic plasticity, and cognitive function (Beckmann et al., 2018) or genes associated with age-related cognitive decline (Ramos-Miguel et al., 2018). Other clinical studies and preclinical drug discovery programs with a focus on synaptic function in AD are now being disclosed. As well as identification of the best novel synaptic therapies, it is equally important that development of synaptic biomarker strategies in living AD patients is also prioritized. While the modality of the most appropriate synaptic biomarker is yet to be determined, it will need to be sensitive enough to detect relatively small longitudinal changes in specific brain regions, and ideally be applicable in both humans and experimental animals to allow translational research opportunities. Future research in this field will determine the exact disease contexts in which synaptic therapies are likely to provide the greatest efficacy. It seems likely that the efficacy of a synaptic therapy will be greatest if it were possible to give it in combination with a disease-modifying treatment. Otherwise, it is likely to be critical to identify how late in the disease process one can still successfully intervene with a synaptic therapy. Nevertheless, the synapse is very likely to represent the critical substrate that links β-amyloid and tau pathologies to the devastating cognitive symptoms of AD.

## Author Contributions

All authors contributed intellectually to the manuscript. JJ and GG wrote the manuscript. All authors approved the manuscript. Figures created with BioRender.

## Conflict of Interest Statement

All authors are employees of Eli Lilly.
